# *Carapa guianensis* Aublet (Andiroba) Seed Oil: Chemical Composition and Antileishmanial Activity of Limonoid-Rich Fractions

**DOI:** 10.1155/2018/5032816

**Published:** 2018-09-06

**Authors:** Iara dos Santos da Silva Oliveira, Carla Junqueira Moragas Tellis, Maria do Socorro dos Santos Chagas, Maria Dutra Behrens, Kátia da Silva Calabrese, Ana Lucia Abreu-Silva, Fernando Almeida-Souza

**Affiliations:** ^1^Rede Nordeste de Biotecnologia, RENORBIO, UFMA, São Luís, Brazil; ^2^Laboratório de Produtos Naturais 5, Farmanguinhos, Fiocruz, Rio de Janeiro, Brazil; ^3^Laboratório de Imunomodulação e Protozoologia, Instituto Oswaldo Cruz, Fiocruz, Rio de Janeiro, Brazil; ^4^Mestrado em Ciência Animal, Universidade Estadual do Maranhão, São Luís, Brazil

## Abstract

Leishmaniasis is a complex of diseases caused by protozoa of the genus* Leishmania* and affects millions of people around the world. Several species of plants are used by traditional communities for the treatment of this disease, among which is* Carapa guianensis* Aubl. (Meliaceae), popularly known as andiroba. The objective of the present work was to conduct a chemical study of* C. guianensis* seed oil and its limonoid-rich fractions, with the aim of identifying its secondary metabolites, particularly the limonoids, in addition to investigating its anti-*Leishmania* potential. The chemical analyses of the* C. guianensis* seed oil and fractions were obtained by electrospray ionization mass spectrometry (ESI-MS). The cytotoxic activity was tested against peritoneal macrophages, and antileishmanial activity was evaluated against promastigotes and intracellular amastigotes of* Leishmania amazonensis*. All the* C. guianensis* seed oil samples analyzed exhibited the same pattern of fatty acids, while the limonoids 7-deacetoxy-7-hydroxygedunin, deacetyldihydrogedunin, deoxygedunin, andirobin, gedunin, 11*β*-hydroxygedunin, 17-glycolyldeoxygedunin, 6*α*-acetoxygedunin, and 6*α*,11*β*-diacetoxygedunin were identified in the limonoid-rich fractions of the oil. The* C. guianensis* seed oil did not exhibit antileishmanial activity, and cytotoxicity was higher than 1000 *μ*g/mL. Three limonoid-rich oil fractions demonstrated activity against promastigotes (IC_50_ of 10.53±0.050, 25.3±0.057, and 56.9±0.043*μ*g/mL) and intracellular amastigotes (IC_50_ of 27.31±0.091, 78.42±0.086, and 352.2±0.145 *μ*g/mL) of* L. amazonensis*, as well as cytotoxicity against peritoneal macrophages (CC_50_ of 78.55±1.406, 139.0±1.523, and 607.7±1.217 *μ*g/mL). The anti-*Leishmania* activity of the limonoid-rich fractions of* C. guianensis* can be attributed to the limonoids 11*β*-hydroxygedunin and 6*α*,11*β*-diacetoxygedunin detected in the chemical analysis.

## 1. Introduction

Leishmaniasis is characterized as zoonotic disease in which humans can be involved in a secondary or accidental manner. They are noncontagious, infectious-parasitic diseases, caused by several species of protozoa of the genus* Leishmania*. Although the efficacy of the drugs available for leishmaniasis depends on which clinical form is being treated and also on the specific geographical location, there remains a need for better therapeutic results for different clinical forms, as well as* Leishmania*-HIV coinfection [1].

Leishmaniasis is classified as neglected disease, and the treatment recommended by the World Health Organization still has many side effects, making patient adherence difficult and contributing to increased cases of resistance to several strains of* Leishmania*. For this reason, new therapeutic alternatives have been sought, including the use of natural products [2].

The use of medicinal plants as an alternative for leishmaniasis treatment is quite common in endemic areas. In an ethnobotanical survey carried out in the northeast of Brazil, forty-nine plant species identified for the topical treatment of skin ulcerations caused by species of the genus* Leishmania *were identified [3]. Herbal medicines are numerous and frequently used, according to the local population, and their practical effectiveness has been observed [4, 5].

Among many medicinal plants in Brazil,* Carapa guianensis* Aublet (andiroba in Portuguese or crabwood in English) offers several benefits to populations resident in the Amazon, due to the medicinal properties of the oil extracted from its seeds, its use in the timber industry, and its ecological value [6]. The* Carapa* (Meliaceae) genus has been described as one of the most used by Amazonian communities for the treatment of leishmaniasis [7].

*C. guianensis* has already demonstrated its effects against protozoa of the genera* Plasmodium* and* Trypanosoma*, due to the limonoids that exist in the oil [8–10]. Limonoids are a structurally diverse series of tetranortriterpenoids found primarily in the Meliaceae family. Much of the research on the biological activities of limonoids has been motivated by the desire to find compounds that are useful for agricultural or medicinal applications [11].

Among the limonoids found in* C. guianensis* seed oil are 6*α*-acetoxygedunin, 7-deacetoxy-7-oxogedunin, andirobin, gedunin and methyl-angolensate [12], guianolides A and B, [13], carapanolides M–S [14], carapanolides C–I [15], carapanolides T-U, and carapanolides V-X [16].

While a study described the activity of* C. guianensis* nanoemulsion on* L. amazonensis* and* L. infantum* [17], the chemical compounds that acted on the parasites were not defined, creating a need for a more detailed investigation of the limonoid-rich fractions of* C. guianensis* seed oil in terms of their leishmanicidal activity. As a result of the medicinal properties already reported for this species, the common treatments used for leishmaniasis in the Amazon region, and the reports in literature, the present study proposed to evaluate the chemical compounds present in the oil and limonoid-rich fractions of* C. guianensis* seed oil and to verify which compounds are involved in their anti-*Leishmania* activity.

## 2. Material and Methods

### 2.1. Collection Area and Obtaining* C. guianensis* Seeds Oil

*C. guianensis* seed oil was obtained from extractive producers in Axixá, Maranhão, Brazil (latitude 02°41′18′′ and 02°56′53′′ south, longitude 44°02′43′′ and 44°09′51′′ west). In relation to the climatic characteristics of the study area, the municipal region of Axixá has an average annual temperature of 26.1°C, while annual rainfall varies from 1,600 to 2,400 mm, and relative air humidity corresponds to about 85%. The collection was carried out in the rainy season in January 2017. Botanical identification was performed out in the Laboratory of Botany and Herbarium of the Universidade Estadual do Maranhão, voucher n° 4.896. The entire process of obtaining* C. guianensis* seed oil was carried out in an artisanal manner. The extraction of the* C. guianensis* seed oil went through the steps of fruit collection, cooking, pulping, and mass treatment for the extraction of oil. After the pulp was removed from the andiroba nuts, the mass was macerated daily to obtain the oil. Five-point oil samples from the same region were collected for chemical analysis. To perform the in vitro experiments, the oils were diluted in DMSO (Sigma, USA) at 200x, the highest tested concentration, with the experiments performed with a maximum concentration of 0.5% of DMSO.

### 2.2. Obtaining of Limonoid-Rich Fractions from* C. guianensis* Seed Oil

*C. guianensis* seed oil (10 g) was fractionated on a silica gel chromatographic column (Merck 60, 0.040-0.063 mm) using a gradient solvent mixture of ethyl acetate in hexane as eluent. The fractions were submitted to chemical analysis by mass spectrometry.

### 2.3. Electrospray Ionization Mass Spectrometry (ESI/MS)

Electrospray ionization mass spectra were obtained using a Q-TOF mass spectrometer (Bruker, Amazon SL-ion trap). To identify the fatty acid profile of* C. guianensis* seed oil, direct infusions were performed from the dilution of 20 *μ*L of the oils in 1 mL of toluene for negative mode (ESI-) analyses. The fractions were submitted to chemical analysis by mass spectrometry after the addition of 800 *μ*L MeOH:H_2_O solution (1:1) with 0.1% formic acid for positive mode (ESI+) and 0.1% NH_4_OH for negative mode (ESI-) analyses. The operating conditions were 3.0-4.0 kV capillary voltage, 100°C temperature source, and cone voltage of 20-40 V. The diluted samples were injected by automatic injection with a continuous flow of 10 *μ*L/min.

### 2.4. Parasite Culture

The isolate of* Leishmania amazonensis* (MHOM/BR/76/MA-76) was maintained in the Laboratório de Imunomodulação e Protozoologia by serial passages in BALB/c mice. Periodically, parasites were isolated from the lesions and maintained in Schneider's Insect Medium (Sigma, USA) supplemented with 10% fetal bovine serum (FBS) (Gibco, USA), penicillin (100 UI/mL), and streptomycin (100 *μ*g/mL) (Sigma, USA) at 26°C in a BOD incubator. To guarantee the infectivity of the promastigote forms, only cultures with a maximum of 6 passages in vitro were used.

### 2.5. Animals

Female BALB/c mice aged 4-6 weeks were obtained from the Instituto de Ciência e Tecnologia em Biomodelos from Instituto Oswaldo Cruz, Rio de Janeiro. All animal experiments were conducted according to the guidelines for experimental procedures of the Fundação Oswaldo Cruz (License L53/2016). During the experiments, all mice were kept at a controlled temperature, receiving food and water* ad libitum*.

### 2.6. Cell Culture

Female BALB/c mice were inoculated with 3 mL 3% sodium thioglycolate (Sigma, USA), and the peritoneal macrophages were harvested with PBS solution after 72 hours. Cells were centrifuged at 1500 rpm and suspended in RPMI 1640 medium (Sigma, USA) supplemented with 10% FBS, penicillin (100 U/mL), and streptomycin (100 *μ*g/mL) at 37°C and 5% CO_2_.

### 2.7. Activity against Promastigote Forms of* L. amazonensis*

Promastigotes of* L. amazonensis* (10^6^ parasites/mL) from a 2-4 day old culture were placed in 96-well plates with different concentrations of oil and limonoid-rich fractions of* C. guianensis* seed oil (3.9 to 500 *μ*g/mL) in a final volume of 200 *μ*L per well for 72 h. They were then incubated in a BOD oven at 26°C. Wells without parasites and wells with only parasites with 1% DMSO were used as controls. The parasite viability was evaluated by the modified colorimetric method based on the tetrazolium dye MTT (3-(4,5-dimethylthiazol-2-yl)-2,5-diphenyltetrazolium bromide) (Sigma, USA) [18]. MTT (5 mg/mL) was added to each well in a volume equal to 10% of the total. After 5 h, 150 *μ*L of DMSO was added to each well to dissolve the formazan crystals. The absorbance was read on a spectrophotometer at a wavelength of 570 nm. Data were normalized according to the formula: % survival = Abs. sample-Abs. blank / Abs. control-Abs. blank × 100. The results were used to calculate IC_50_ (50% inhibition of parasite growth) by nonlinear regression with GraphPad Prism 6.0. Amphotericin B was used as a reference drug.

### 2.8. Cytotoxicity Assay

Peritoneal murine macrophages cultured in RPMI medium were pipetted into 96-well plates at a density of 10^5^ cells/mL. After 24 hours of incubation at 37°C in 5% CO_2_, the culture medium from each well was withdrawn, supplemented with different dilutions of the oil or limonoid-rich fractions of* C. guianensis* seed oil (7.8 to 1000 *μ*g/mL), and incubated again. As a control, wells were maintained with only 1% DMSO cells or with medium only. After 24 hours, cell viability was measured by MTT colorimetric assay as previously described [19].

### 2.9. Activity against Intracellular Amastigotes of* L. amazonensis* and Selectivity Index (SI)

Peritoneal macrophages were cultured in 24-well plates (1 mL/well; 5×10^5^ cells/mL) containing glass coverslips and incubated for two hours at 37°C and 5% CO_2_. Nonadherent macrophages were removed and promastigote forms of* L. amazonensis* (5x10^6^ cells/mL) were added to each well for 24 hours at 34°C and 5% CO_2_. Noninternalized promastigotes were removed via PBS lavage and the cells were treated for 24 h with limonoid-rich fractions of* C. guianensis* seed oil (LF3: 5 to 20 *μ*g/mL; LF4: 6.25 to 100 *μ*g/mL; LF5: 25 to 200 *μ*g/mL) or amphotericin (0.5 to 4 *μ*g/mL). Wells without treatment were kept as controls. The coverslips with cells were then fixed with Bouin solution, stained with Giemsa (Merck, USA), and examined by light microscopy. IC_50_ was calculated with the GraphPad Prism 6.0 software package from the nonlinear regression curve of the concentration log of the limonoid-rich fractions of the oil by the normalized response of the number of intracellular amastigotes in 200 cells. The percentage of infected cells was obtained from the number of infected cells divided by two. The mean number of amastigotes per cell was obtained from the number of intracellular amastigotes in 200 cells divided by the number of infected cells. The selectivity index was calculated from the ratio of CC_50_ versus the IC_50_ for intracellular amastigotes.

### 2.10. Statistical Analysis

The numerical results were expressed as mean ± standard deviation and were organized into tables or plotted in graphs. IC_50_ and CC_50_ were calculated from the nonlinear regression curve from the log of the inhibitor concentration versus the normalized response. Data were statistically analyzed by the Kruskal-Wallis test and Dunn's multiple comparison test and differences were considered significant when p<0.05. All analyses were performed with GraphPad Prism 6 software (GraphPad Software Inc.).

## 3. Results

### 3.1. Phytochemical Profile of* C. guianensis* Seed Oils

**Figure 1:**
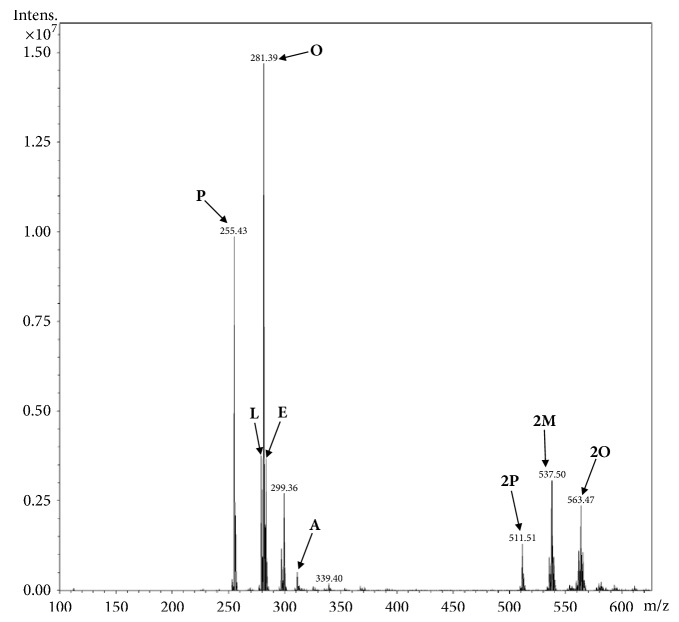
Mass spectra of the* Carapa guianensis* seed oil in negative mode. Oil samples A1 to A5. P: palmitic acid; L: linoleic acid; O: oleic acid; E: stearic acid; A: arachidic acid; 2P: palmitic acid dimer; 2M: dimer of margaric acid; 2O: dimer of oleic acid.

The five samples of* C. guianensis* seed oil had their phytochemical profiles compared by mass spectrometry using the direct infusion technique (ESI/MS).  Figure 1 shows the mass spectra obtained for the oils in the negative mode, with the presence of molecular ions m/z 255, 279, 281, 283, and 311 corresponding to the deprotonated ions [M-H] of the palmitic, linoleic, oleic, stearic, and arachidic acids, respectively [20]. The presence of the ions in m/z 511, 537, and 563 characterizes the dimeric forms [2M-H] of palmitc, margaric and oleic acids, respectively. The comparison of the spectra revealed that the composition of free and dimeric fatty acids is very similar to the 5 types of oils analyzed, being altered only, even if just a little, in the intensity of some ions, which may indicate a small variation in the concentrations of these free and dimeric fatty acids.

### 3.2. Chemical Analysis of Limonoid-Rich Fractions of* C. guianensis* Seed Oil

**Figure 2:**
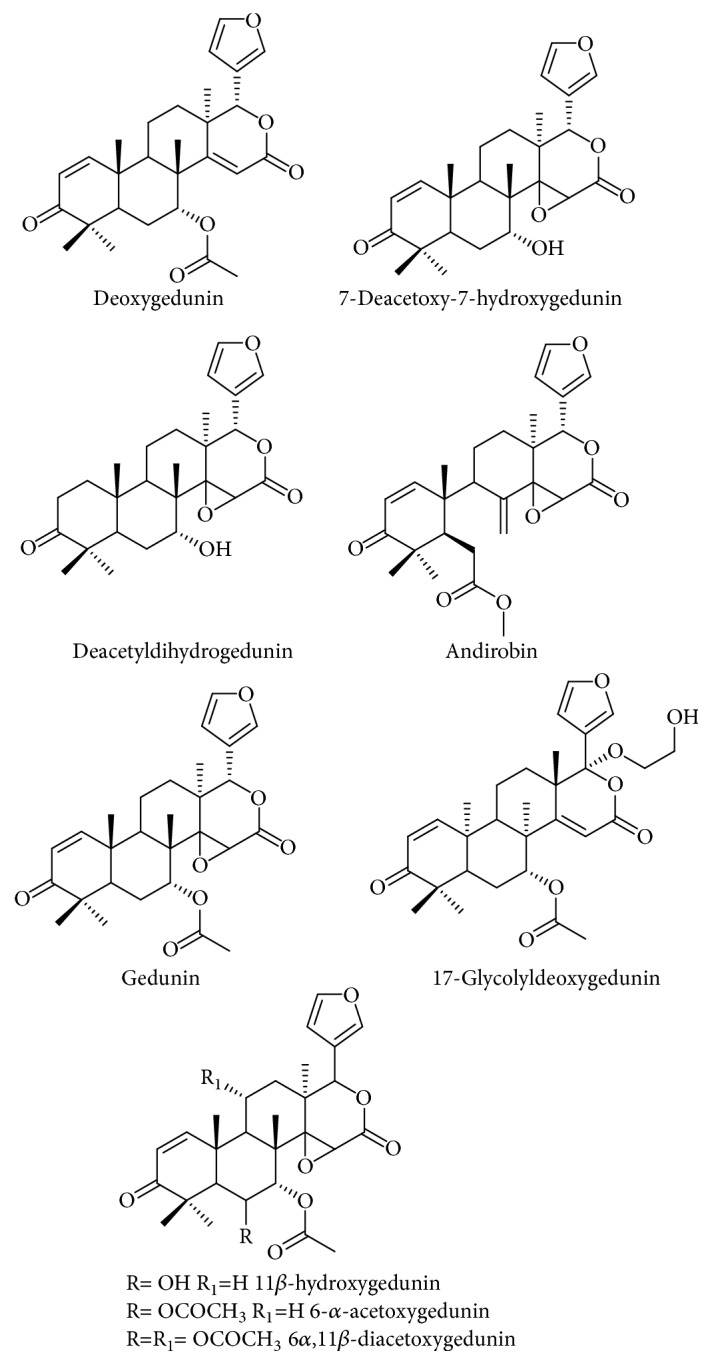
Chemical structure of limonoids identified in limonoid-rich fraction of* Carapa guianensis* seeds oil.

**Figure 3:**
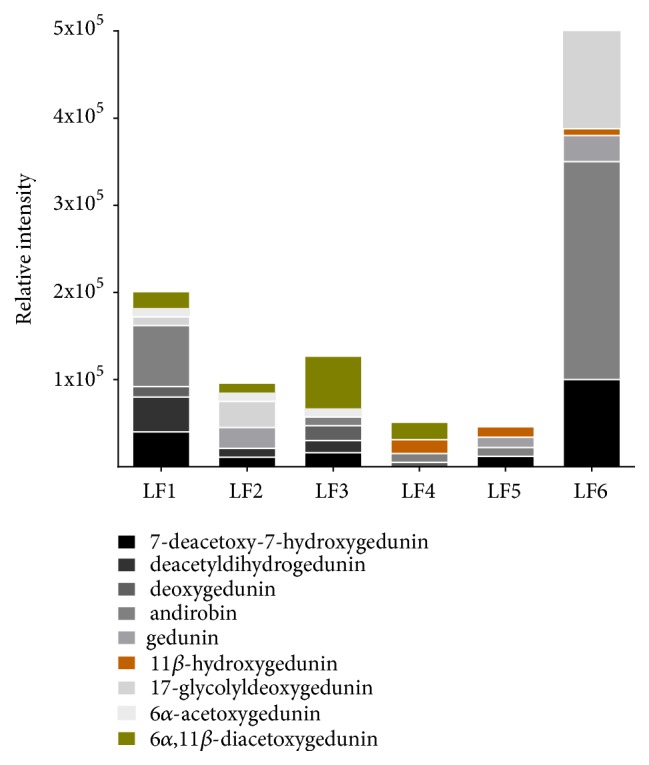
Limonoid composition and relative intensity of limonoid-rich fractions of* Carapa guianensis* seed oil.

*C. guianensis* seed oil was fractionated by silica gel column chromatography using hexane and graphed ethyl acetate and yielded six limonoid enriched fractions (LF1 to LF6). The limonoids identified in limonoid-rich fractions, with their respective molecular ions (m/z), were 7-deacetoxy-7-hydroxygedunin (441), deacetyldihydrogedunin (443), deoxygedunin (467), andirobin (469), gedunin (483), 11*β*-hydroxygedunin (499), 17-glycolyldeoxygedunin (527), 6*α*-acetoxygedunin (541), and 6*α*,11*β*-diacetoxygedunin (599) (Figure 2) [20–23]. The composition of each limonoid-rich fraction showed that the fractions LF1, LF2, and LF3 presented the greatest variability of limonoids, while LF6 presented the greatest amount of limonoids between them. The limonoids with the highest relative intensity were andirobin and 17-glycolyldeoxygedunin (Figure 3).

### 3.3. Activity against Promastigote and Cytotoxicity of Oil and Limonoids-Rich Fractions of* C. guianensis* Seed Oil

*C. guianensis* seed oil did not exhibit leishmanicidal activity against* L. amazonensis* promastigote forms and did not present cytotoxicity even in the highest analyzed concentrations, 500 and 1000 *μ*g/mL, respectively. On the other hand, the limonoid-rich fractions LF3, LF4, and LF5 exhibited leishmanicidal activity, with fraction LF3 and LF5 presenting the lowest and highest IC_50_ value among them. The cytotoxicity of limonoid-rich fractions LF3, LF4, and LF5 demonstrated the same pattern of activity against promastigotes, with the CC_50_ increasing from LF3 to LF5. The fractions LF1, LF2, and LF6 did not exhibit activity against promastigote forms or cytotoxicity at the analyzed concentrations. Amphotericin had IC_50_ for promastigote and CC_50_ values similar to those described in literature (Table 1).

### 3.4. Activity against Intracellular Amastigote and SI of Limonoid-Rich Fractions of* C. guianensis* Seeds Oil

**Figure 4:**
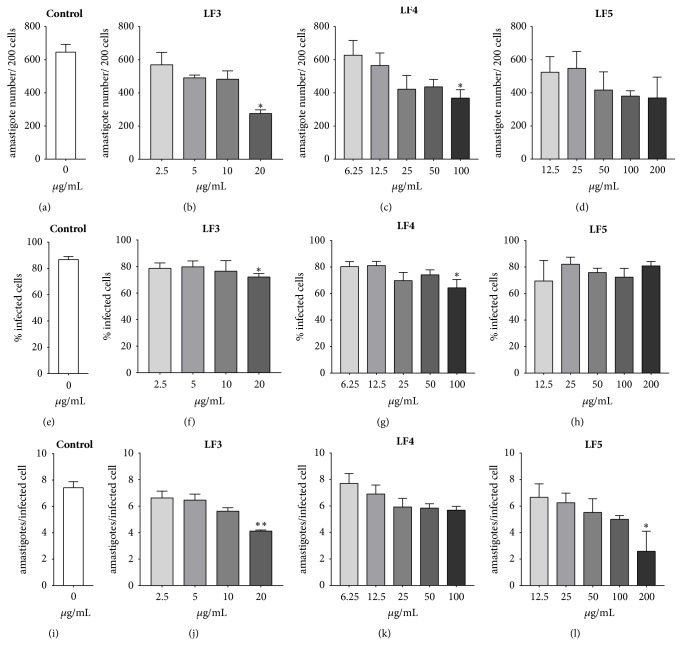
Infection parameters of BALB/c peritoneal macrophages infected with* Leishmania amazonensis* and treated with limonoid-rich fractions of* Carapa guianensis* seed oil. LF: limonoid-rich fraction. Data represent mean ± standard deviation of two independent experiments realized in quadruplicate. *∗*p<0.05 and *∗∗*p<0.01 when compared with the untreated group (Control) by Kruskal-Wallis followed by Dunn's multiple comparisons test.

**Figure 5:**
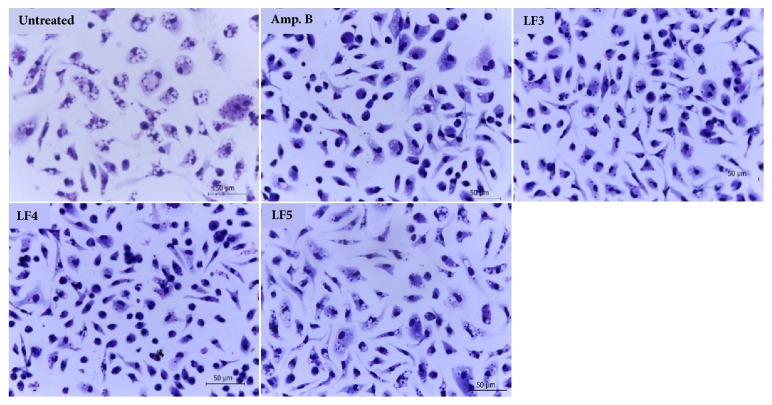
Light microscopy of BALB/c peritoneal macrophages infected with* Leishmania amazonensis* and treated with limonoids-rich fractions of* Carapa guianensis* seed oil FL3 (20*μ*g/mL), FL4 (100*μ*g/mL), FL5 (200*μ*g/mL), or amphotericin B (4*μ*g/mL). Giemsa, 100x oil objective. The images are representative of two independent experiments performed in quadruplicate.

The same limonoid-rich fractions that were active against promastigotes also exhibited activity against intracellular amastigote forms, although they had higher IC_50_ values than those obtained for the promastigote forms. The intracellular amastigote IC_50_ values for LF3, LF4, and LF5 ranged from 27.31±0.091 to 352.2±0.180 *μ*g/mL, with fraction LF3 having the lowest result of the three. The selectivity index was also higher for LF3 than for LF4 and LF5 (Table 1). Analysis of the parameters of infection showed that LF3 had the most consistent activity against the intracellular amastigote, inducing a reduction in the number of amastigotes per 200 cells, the percentage of infected cells, and the mean amastigotes per cell at 20 *μ*g/mL. LF4 decreased the number of amastigotes per 200 cells and the percentage of infected cells at 100 *μ*g/mL, while LF4 only decreased the mean number of amastigotes per cell at 200 *μ*g/mL (Figure 4). A reduction in intracellular amastigotes in peritoneal macrophages infected with* L. amazonensis* and treated with LF3, LF4, and LF5 at these concentrations was clearly observed with light microscopy (Figure 5).

## 4. Discussion

The present study investigated the effect of oil and limonoid-rich fractions of* C. guianensis* seed oil on promastigotes and intracellular amastigotes of* L. amazonensis*. The chemical constituents of the* C. guianensis* seed oil involved in the activity and cytotoxicity against BALB/c peritoneal macrophages were also identified.

Different combinations of solvents and parts of plant may have different biological effects due to the variety and relative concentrations of compounds present in the final extract. Information on solvents, plant tissues, and methods of preparation and extraction is therefore important for standardization between studies. Unfortunately, studies that report the composition of vegetal material and associate the same with biological activity are scarce, especially in the Amazon region [24].

According to our results,* C. guianensis* seed oil exhibited anti-*Leishmania* activity higher than 500 *μ*g/mL for the promastigote form after 72 hours of treatment. A recent study with* C. guianensis* seed oil nanoemulsion revealed activity against promastigotes after 48 hours of treatment, with an IC_50_ of 260±29 *μ*g/mL [17]. Although the oil used to prepare the nanoemulsion is also of Amazonian origin, as in the present study, only classes of chemical compounds such as limonoids and the fatty acids palmitic oil, oleic acid, and linoleic acid have been described in oil that originated the nanoemulsion. In addition, nanoemulsion may facilitate the entry of* C. guianensis* seed oil compounds and favored leishmanicidal activity. It is noteworthy that* C. guianensis* seed oil used in our experiments did not also demonstrate in vitro cytotoxicity, a characteristic that was not evaluated in nanoemulsion.

The 6 limonoid-rich fractions obtained from the* C. guianensis* seed oil were analyzed for antileishmanial activity and the chemical constituents in each were identified. The LF1, LF2, and LF6 fractions had an IC_50_ higher than 500 *μ*g/mL, similar to the oil. The LF3, LF4, and LF5 fractions were able to decrease the IC_50_ in comparison with the results of the* C. guianensis* seed oil, both for the promastigote forms and the intracellular amastigotes, demonstrating the potential of limonoids of the gedunin group, especially 11*β*-hydroxygedunin 6*α*,11*β*-diacetoxygedunin, which was more concentrated in these fractions. It was found that the activity of the limonoid-rich fractions against the promastigote forms was more effective than against the intracellular amastigote forms. In contrast,* C. guianensis* nanoemulsion oil exhibited better results against amastigote forms [17]. When treating* L. amazonensis*-infected macrophages with andiroba nanoemulsion for 24 hours (200-300 *μ*g/mL), it significantly reduced* L. amazonensis* infection levels in macrophages (54% and 96%, respectively); however the chemicals involved in this activity could not be identified.

The plant* C. guianensis* belongs to the Meliaceae family, which is chemically distinguished by its production of limonoids, highly oxygenated tetranortriterpenoid compounds with a wide range of biological activities [11]. Compounds of this type are found in only four other families of the plant kingdom: Rutaceae, Cneoraceae, Simaroubaceae, and Ptaeroxylaceae where 1,300 limonoids have already been described, with more than 35 different carbon structures [11, 25–27].* C. guianensis* seed oil is composed of palmitic, oleic, and linoleic acid, in addition to an unsaponifiable fraction composed mainly of limonoids, which are probably responsible for their biological activity [28].

According to the classification that covers the majority of the limonoids isolated from the Meliaceae family, these can be divided into main groups, according to the carbon skeleton pattern presented [25, 29, 30]. The present study was able to identify previously undescribed limonoids for* C. guianensis* seed oil belonging to the gedunin group, such as the 17-glycolyldeoxygedunin. The limonoids found in the oil fractions were part of the andirobin and gedunin group. These chemical compounds are probably involved in the profile of leishmanicidal activity observed in the fractions evaluated.

Limonoids of different plant species have already demonstrated antiparasitic activity [31, 32]. Extracts and fractions rich in limonoids have already demonstrated activity on promastigotes and amastigotes of* L amazonensis*, such as* Azadirachta indica* A. Juss. [33], a representative of the Meliaceae family that has already had its limonoids isolated, such as gedunin and nimbolide [10, 34].

The LF3 and LF4 fractions, respectively, had better leishmanicidal activity, probably due to the presence of the 11*β*-hydroxygedunin and 6*α*,11*β*-diacetoxygedunin limonoids. A compound that may have influenced the activity of the LF3 fraction is the 6*α*,11*β*-diacetoxygedunin, which was absent in the LF5 fraction and present in low amounts in LF4 fraction. The presence of 11*β*-hydroxygedunin in LF5 and the concomitant presence of 11*β*-hydroxygedunin and 6*α*,11*β*-diacetoxygedunin in LF4 may be the responsible for their activities. The LF5 fraction had better activity against the promastigote form, demonstrating a significant activity reduction against the intracellular amastigote and low cytotoxicity against peritoneal macrophages. It was necessary to increase the concentration by up to 6 times in comparison with the promastigote form, revealing low permeability and diffusion through cell membrane. The limonoid structure may be related to the potential for activity and may in some situations make them more or less active. Some groups of limonoids may be two to three times more active than other limonoids [35].

Treatment with the LF3, LF4, and LF5 fractions was able to reduce infection levels in* L. amazonensis* cells. However, they also showed cytotoxicity, meaning caution is required with the use of limonoid-rich fractions from* C. guianensis* seed oil. Certain rings in the limonoid nucleus may be critical to the activity of these compounds. Alterations in ring A of the limonoid nucleus may lead to a loss of cytotoxic activity for neoplasic cells, for example, while changes in the D ring can be tolerated without any apparent loss of activity [36]. In this way, assays with the fractions in the nanoemulsion form as a carrier system are likely to improve the specificity of the chemical compounds against the* L. amazonensis* parasite. These results demonstrated that the limonoid-rich fractions of* C. guianensis* seeds oil were able to promote leishmanial activity. However, more studies should focus on the detailed characterization, quantification, and projection of a versatile and simple natural or synthetic limonoid route.

## 5. Conclusions

The analysis of the mass spectra obtained from the oil allowed the identification of fractions rich in limonoids derived from* C. guianensis* seed oil. Limonoid-rich fractions showed leishmanicidal activity against the promastigotes and amastigotes of* L. amazonensis*. The anti-*Leishmania* activity was assigned to the limonoids 11*β*-hydroxygedunin and 6*α*,11*β*-diacetoxygedunin, identified in the active limonoid-rich fractions of* C. guianensis* seed oil.

## Figures and Tables

**Table 1:** Activity against the promastigotes and intracellular amastigotes of *Leishmania amazonensis*, cytotoxicity in BALB/c peritoneal macrophages, and selectivity index of oil and limonoid-rich fractions of *Carapa guianensis* seed oil.

Compounds	IC_50_ (*μ*g/mL) promastigote	CC_50_ (*μ*g/mL) peritoneal macrophage	IC_50_ (*μ*g/mL) intracellular amastigote	IS
*C. guianensis* seed oil	>500	>1000	–	–
LF1	>500	>1000	–	–
LF2	>500	489.4 ± 0.173	–	–
LF3	10.53 ± 0.050	78.55 ± 1.406	27.31 ± 0.091	2.87
LF4	25.30 ± 0.057	139.0 ± 1.523	78.42 ± 0.086	1.77
LF5	56.90 ± 0.043	607.70 ± 1.217	352.2 ± 0.145	1.72
LF6	>500	>1000	–	–
Amphotericin B	1.37±0.124	6.26±0.286	0.2 ± 0.180	31.3

IC_50_: inhibitory concentration of 50% parasites. CC_50_: cytotoxicity concentration of 50% cells. SI: selectivity index calculated from the ratio of CC_50_ versus the IC_50_ for intracellular amastigotes. LF: limonoid-rich fraction of *C. guianensis* seed oil. Data represent mean ± standard deviation of three experiments performed at least in triplicate.

## Data Availability

The data used to support the findings of this study are included within the article.

## Abbreviations

CC_50_: 50% cytotoxic concentration
Abs: Absorbance
DMSO: Dimethyl sulfoxide
ESI/MS: Electrospray ionization mass spectrometry
ESI+: Positive mode ionization mass spectrometry
ESI-: Negative mode ionization mass spectrometry
FBS: Fetal bovine serum
HIV: Human immunodeficiency virus
IC_50_: Inhibitory concentration of 50%
SI: Selectivity index
LF: Limonoids fraction
MTT: 3-(4,5-Dimethylthiazol-2-yl)-2,5-diphenyltetrazolium bromide
M-H+: Deprotonated ions
m/z: Mass-to-charge ratio
PBS: Phosphate buffered saline.
